# Intrasheath Peroneal Tendon Subluxation: A Report of Three Cases with Technical Note

**DOI:** 10.1055/s-0044-1779310

**Published:** 2024-06-03

**Authors:** Raquel Lima Cunha, Alexandre Castro, Pedro Atilano Carvalho, Marta Gomes, António Miranda, João Teixeira

**Affiliations:** 1 Departamento de Ortopedia e Traumatologia, Centro Hospitalar Entre o Douro e Vouga, Santa Maria da Feira, Portugal; 2 Departamento de Ortopedia e Traumatologia, Hospital da Luz Arrábida, Porto Portugal, Vila Nova de Gaia, Portugal

**Keywords:** ankle injuries/diagnostic imaging, joint instability, tendons/diagnostic imaging, tendon injuries/diagnostic imaging, ultrasonography

## Abstract

Peroneal intrasheath instability is a painful popping sensation and audible clicking of the lateral ankle. This condition is not commonly reported, and its exact incidence remains unknown. It consists of a transient retromalleolar subluxation of the peroneal tendons, with an abnormal motion of the peroneal tendons relative to each other, with the superior peroneal retinaculum intact. Diagnosis requires high clinical suspicion and dynamic ultrasound is the best imaging study to evaluate peroneal instability when the superior peroneal retinaculum is intact, for diagnosing peroneal intrasheath instability. The goal of the present study is to describe how to reach the diagnosis and to describe and evaluate the surgical technique for the treatment of this pathology. In the present report, we describe three cases of this pathology that received successful surgical treatment with peroneal groove-deepening procedure and retinaculoplasty of the superior retinaculum. This surgical technique provides good outcomes when conservative treatment fails.

## Introduction

Recurrent peroneal tendon subluxation is an uncommon and disabling injury. 1 Monteggia was the first to describe this lesion, in a ballet dancer. 1 2 The mechanism of the acute subluxation is usually by dorsiflexion or inversion of the foot with peroneal muscles strongly contracted. 3 This pathology is often mistaken for an ankle sprain, that is why peroneal tendon lesions, especially subluxations, are underdiagnosed. 3 More rarely, peroneal intrasheath instability may develop, consisting of a transient retromalleolar subluxation of the peroneal tendons, with an abnormal motion of the peroneal tendons relative to each other 4 5 6 7 In this case, there is no disruption of the Superior Peroneal Retinaculum (SPR). 4 5 6 This type is commonly missed on a physical examination because there is no override of the tendons on the lateral malleolus during subluxation. 5 7

Clinical findings include lateral ankle pain and a transient popping sensation during contraction of the peroneal muscle. 6 Patients may report an audible click during active dorsiflexion of the ankle without evident visible displacement of the peroneal tendons over the lateral malleolus. 5 6

**Fig. 1:**
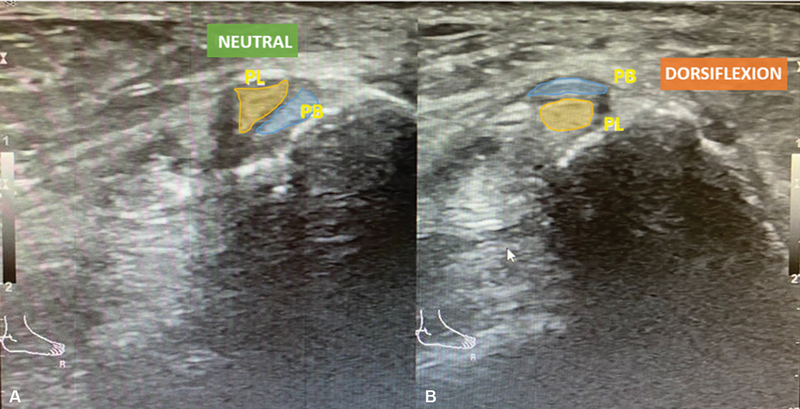
Diagnostic dynamic ultrasound of the lateral ankle showing normal retromalleolar position of peroneal tendons at rest ( **A** ) and intrasheath subluxation of in dorsiflexion ( **B** ). Abbreviations: PL, peroneal longus tendon; PB, peroneal brevis tendon.

**Fig. 2:**
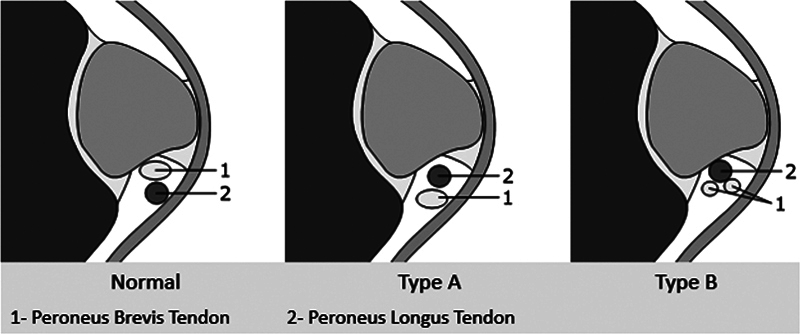
Classification of peroneal intrasheath subluxation.

Magnetic resonance imaging (MRI) is often performed for differential diagnosis of painful lateral ankle but is commonly described as normal. 3 8 Dynamic ultrasound (DUS) and comparative assessment is crucial for evaluating this condition, showing the abnormal motion of peroneal tendons relative to each other ( Fig. 1 ) within an intact SPR. 3 5 Furthermore, DUS allows differentiation between type A intrasheath instability (there is a relative switching of the anatomical alignment of tendons) and type B intrasheath instability (the peroneus brevis tendon has a longitudinal split tear through which the peroneus longus subluxate) ( Fig. 2 ). 5 6 In 1987, McConkey et al. 4 have proposed a surgical treatment with retinaculoplasty of the superior retinaculum, without peroneal groove-deepening procedure. 4

In the present article, we describe the case report of three patients treated for intrasheath peroneal tendon subluxation.

## Case Description

### Case 1

A 39-year-old woman with history of previous fixation of right lateral malleolus fracture had the implants removed and was submitted to a Broström-Gould procedure due to lateral instability of the right ankle. Afterwards, the patient developed a popping sensation and audible clicking localized to the peroneal tendons, especially during dorsiflexion, associated with lateral ankle pain. Physical examination revealed an audible click upon dorsiflexion of the ankle, but no tendon dislocation over the lateral malleolus was observed. An MRI was ordered and showed no peroneal tendon rupture and an intact SPR. The correct diagnosis was made only after DUS revealed an intrasheath dislocation of the peroneal tendons ( Fig. 1 ). This patient first underwent conservative treatment with physical therapy and after failure of the conservative treatment, was submitted to surgical treatment.

### Case 2

A 21-year-old woman with previous history of ankle sprain consulted for a painful popping sensation and audible clicking in dorsiflexion. During physical examination, there was an audible click upon dorsiflexion. A DUS showed intrasheath subluxation of the peroneal tendons in dorsiflexion, with intact peroneal tendons and SPR. This patient also underwent conservative treatment which failed to relieve symptoms and was then submitted to surgical treatment.

### Case 3

A 20-year-old woman with no medical record consulted for a painful popping sensation, audible clicking in dorsiflexion. Physical examination revealed an audible click upon dorsiflexion of the ankle. A DUS showed intrasheath subluxation of the peroneal tendons in dorsiflexion, with intact SPR and no ruptures of the peroneal tendons confirmed with MRI. This patient underwent conservative treatment with analgesics and physiotherapy and was then submitted to surgical treatment.

## Surgical Technique

**Fig. 3:**
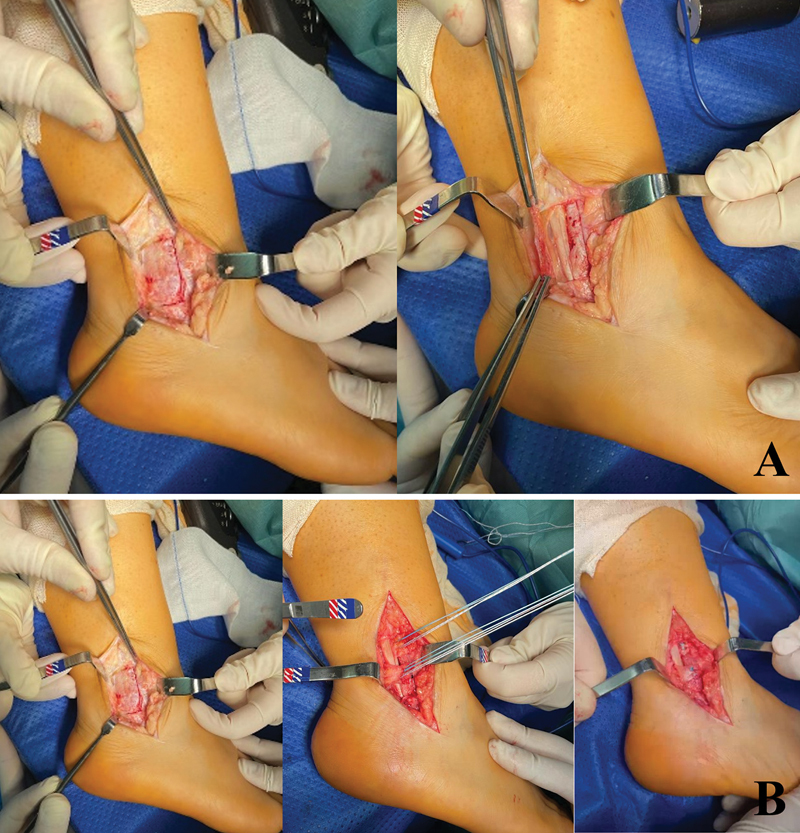
( **A** ) Approach: Posterolateral incision with visualization of the entire superior peroneal retinaculum (left) and reflected peroneal retinaculum (right). ( **B** ) Retinaculoplasty: the superior peroneal retinaculum is exposed (1) and then divided in two flaps (2). The proximal flap is placed between the two peroneal tendons and the distal flap is placed superficial to the peroneal tendons in a native position (3).

**Fig. 4:**
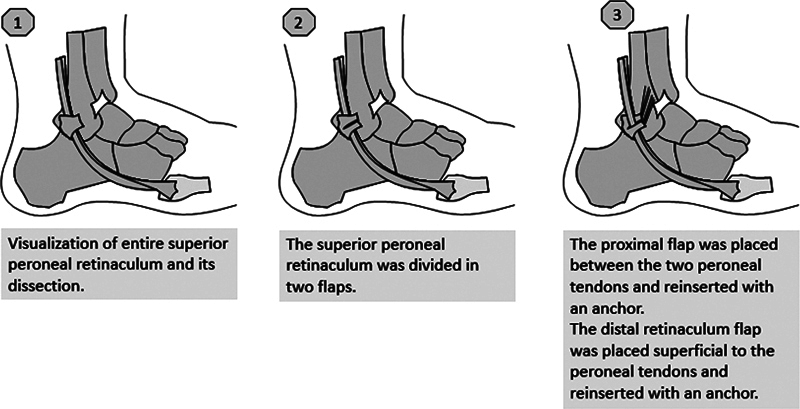
Diagram of the surgical technique Diagram.

The patient is placed in lateral decubitus position and a posterolateral approach to the peroneal tendons is performed. The entire superior peroneal retinaculum is visualized, dissected, and then posteriorly reflected from its malleolar insertion ( Fig. 3 and 4 ).

A deepening of the peroneal groove is conducted, preserving the posterior fibrocartilage gliding surface. The SPR is then divided in two flaps. The proximal flap is placed between the two peroneal tendons, to prevent intrasheath dislocation, and reinserted with an anchor. The distal retinaculum flap is placed superficial to the peroneal tendons and reinserted in its native position to avoid dislocation of the peroneal tendons over the lateral malleolus ( Fig. 3 and 4 ).

## Outcomes

At the end of the surgical procedure, congruence, mobility, and stability must be verified. The patient is immobilized with a plaster cast for 3 weeks and subsequent immobilization with a walker boot for 3 weeks, with progressive weight bearing allowed.

**Fig. 5:**
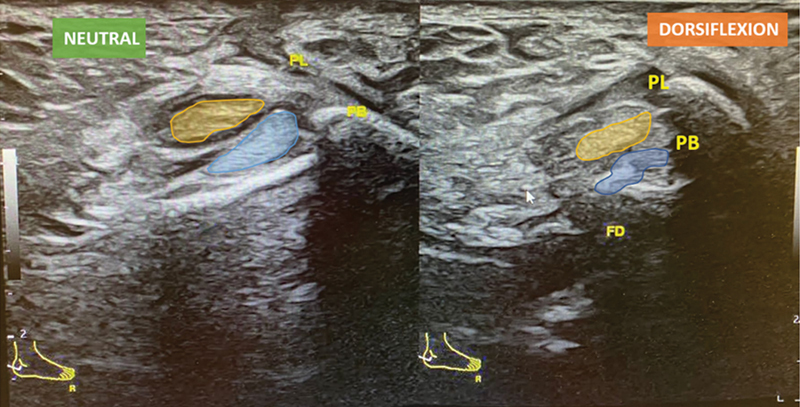
Postoperative dynamic ultrasound: normal retromalleolar positioning of the peroneal tendons both at rest (left) and in dorsiflexion (right). Abbreviations: PL, peroneal longus tendon; PB, peroneal brevis tendon.

Patients were evaluated at 2 weeks and then at 1, 3, 6 and 12 months postoperatively. In the last evaluation, at 1-year postoperatively, all patients were satisfied and no longer presented symptoms of intrasheath peroneal subluxation. Postoperative DUS ultrasound showed normal retromalleolar positioning of the peroneal tendons both at rest and in dorsiflexion ( Fig. 5 ). However, one patient (Case 2) remains with slighter retromalleolar pain and the other (Case 1) complains of ankle pain (probably a sequela of the fracture).

## Discussion

Peroneal intrasheath instability is not commonly reported, and its exact incidence remains unknown. 5 Post-traumatic changes can affect the SPR or the peroneal groove. 7 Patients more often have a history of multiple inversion ankle injuries. 7 In Case 1, the patient has an instability requiring surgical treatment, which predisposed the patient to repeated ankle sprains due to the inversion mechanism. This may have contributed to the consequent instability of the peroneal tendons. In Case 2, the patient has a history of recurrent ankle sprains that may also have contributed to this injury. In all cases, the patients had a dislocation of the peroneal tendons observed in the DUS evaluation, without any rupture identified neither in the ultrasound evaluation nor in the MRI evaluation, so it is classified as a type A instability.

Mostly, MRI is useful to evaluate anatomical variations or intratendinous abnormalities such as tears of tendons. 3 6 The DUS is the best imaging study because it allows the examination of tendons during their physiologic range of motion. 5 This provides a more accurate evaluation of what occurs when patients experience subluxation symptoms. 6 8

Some studies argue that intrasheath instability of the peroneal tendons results from several factors that lead to an effective narrowing of the fibro-osseous tunnel. Thus, surgical procedures should be aimed at restoring sufficient volume of this anatomic area as well as reconstruct the SPR to prevent both intrasheath subluxation and anterior dislocation of peroneal tendons. 4 6

In this technique, posterior deepening of the peroneal groove was performed to increase the depth and surface area and superior retinaculoplasty was performed to prevent peroneal tendons from dislocating with each other or anteriorly.

## Conclusions

Dynamic ultrasound is essential and may reveal an otherwise undiagnosed intrasheath subluxation of the peroneal tendons. Groove-deepening with retinaculoplasty of superior peroneal retinaculum seems to be a successful procedure in these symptomatic patients.
