# Supplementary material for: Intrasheath Peroneal Tendon Subluxation: A Report of Three Cases with Technical Note
Source: Rev Bras Ortop (Sao Paulo). 2024 Jun 3;59(6):e1003–7. doi: 10.1055/s-0044-1779310 (PMC11663060; doi:10.1055/s-0044-1779310)
Supplement: Related texts

# Research article: Subluxação intrabainha dos tendões fibulares: Relato de três casos com nota técnica

**Keywords:** instabilidade articular, tendões/diagnóstico por imagem, traumatismos do tornozelo/diagnóstico por imagem, traumatismos dos tendões/diagnóstico por imagem, ultrassonografia

## Abstract

A instabilidade intrabainha fibular provoca dor com estalo e clique audível na lateral do tornozelo. Esta doença é pouco relatada e sua incidência exata é desconhecida. Consiste em uma subluxação retromaleolar transitória dos tendões fibulares com movimento anormal dos tendões fibulares entre si e integridade do retináculo fibular superior. O diagnóstico requer alta suspeita clínica e a ultrassonografia dinâmica é o melhor estudo de imagem para avaliação da instabilidade fibular quando o retináculo fibular superior está intacto para detecção de instabilidade intrabainha fibular. O objetivo do presente estudo é descrever como chegar ao diagnóstico e relatar e avaliar a técnica cirúrgica para o tratamento desta doença. Neste relato, descrevemos três casos da doença que foram submetidos ao tratamento cirúrgico com sucesso com procedimento de aprofundamento do sulco fibular e retinuloplastia do retináculo superior. Esta técnica cirúrgica proporciona bons resultados em caso de falha do tratamento conservador.

## Introdução

A subluxação recorrente do tendão fibular é uma lesão incomum e incapacitante. 1 Monteggia foi o primeiro a descrever essa lesão, em uma bailarina. 1 2 O mecanismo da subluxação aguda geralmente é a dorsiflexão ou inversão do pé com forte contração dos músculos fibulares. 3 A doença é muitas vezes confundida com uma entorse de tornozelo, por isso as lesões do tendão fibular, em especial as subluxações, são subdiagnosticadas. 3 Com maior raridade, pode haver instabilidade intrabainha fibular, com subluxação retromaleolar transitória dos tendões fibulares e movimento anormal dos tendões fibulares entre si. 4 5 6 7 Nesse caso, não há interrupção do retináculo fibular superior. 4 5 6 Esse tipo de lesão geralmente passa despercebido no exame físico porque não há sobreposição dos tendões do maléolo lateral durante a subluxação. 5 7

Os achados clínicos incluem dor no aspecto lateral do tornozelo e uma sensação transitória de estalo durante a contração do músculo fibular. 6 Os pacientes podem relatar um clique audível durante a dorsiflexão ativa do tornozelo sem deslocamento visível dos tendões fibulares sobre o maléolo lateral. 5 6

A ressonância magnética (RM) é bastante realizada para diagnóstico diferencial da dor no aspecto lateral do tornozelo, mas seus achados são comumente descritos como normais. 3 8 A ultrassonografia dinâmica (USD) e a avaliação comparativa são cruciais para o diagnóstico da doença, mostrando o movimento anormal dos tendões fibulares em relação uns aos outros ( Fig. 1 ) dentro de um retináculo fibular superior intacto. 3 5 Além disso, a USD permite a diferenciação da instabilidade intrabainha de tipo A (em que há uma mudança relativa no alinhamento anatômico dos tendões) e tipo B (em que o tendão fibular curto sofre uma ruptura longitudinal pela qual há subluxação do fibular longo) ( Fig. 2 ). 5 6 Em 1987, McConkey et al. 4 propuseram o tratamento cirúrgico com retinuloplastia do retináculo superior, sem procedimento de aprofundamento do sulco fibular. 4

**Fig. 1:** Ultrassonografia dinâmica diagnóstica do aspecto lateral do tornozelo mostrando a posição retromaleolar normal dos tendões fibulares em repouso ( **A** ) e subluxação intrabainha em dorsiflexão ( **B** ). Abreviaturas: PL, tendão fibular longo; PC, tendão fibular curto.

**Fig. 2:** Classificação da subluxação intrabainha fibular.

Relatamos aqui os casos de três pacientes submetidos a tratamento por subluxação intrabainha do tendão fibular.

## Descrição dos Casos

## Caso 1

Uma mulher de 39 anos com histórico de fixação prévia de fratura do maléolo lateral direito foi submetida à remoção dos implantes e à cirurgia de Broström-Gould devido a instabilidade do aspecto lateral do tornozelo direito. Depois, a paciente desenvolveu sensação de estalo e clique audível localizado nos tendões fibulares, principalmente durante a dorsiflexão, associados à dor no aspecto lateral do tornozelo. O exame físico revelou um clique audível durante a dorsiflexão do tornozelo, mas não foi observada luxação do tendão sobre o maléolo lateral. Uma RM foi solicitada e revelou ausência de ruptura dos tendões fibulares e integridade do retináculo fibular superior. O diagnóstico correto foi feito somente depois que a USD mostrou uma luxação intrabainha dos tendões fibulares ( Fig. 1 ). A princípio, esta paciente recebeu tratamento conservador com fisioterapia e, devido ao insucesso terapêutico, foi submetida à cirurgia.

## Caso 2

Uma mulher de 21 anos com história prévia de entorse de tornozelo foi atendida por sensação dolorosa de estalo e clique audível em dorsiflexão. Durante o exame físico, um clique audível durante a dorsiflexão foi percebido. A USD mostrou subluxação intrabainha dos tendões fibulares em dorsiflexão, com tendões fibulares e retináculo fibular superior intactos. Esta paciente também recebeu tratamento conservador, que não levou ao alívio dos sintomas, e, então, foi submetida à cirurgia.

## Caso 3

Uma mulher de 20 anos sem histórico médico foi atendida por sensação dolorosa de estalo e clique audível em dorsiflexão. O exame físico revelou um clique audível durante a dorsiflexão do tornozelo. A USD mostrou subluxação intrabainha dos tendões fibulares em dorsiflexão, com retináculo fibular superior intacto e ausência de rupturas dos tendões fibulares confirmados à RM. Esta paciente recebeu tratamento conservador com analgésicos e fisioterapia e, posteriormente, foi submetida à cirurgia.

## Técnica Cirúrgica

O paciente é colocado em decúbito lateral e é realizada uma abordagem posterolateral dos tendões fibulares. Todo o retináculo fibular superior é visualizado, dissecado e posteriormente refletido a partir de sua inserção maleolar ( Figs. 3 e 4 ).

**Fig. 3:** ( **A** ) Abordagem: incisão posterolateral com visualização de todo o retináculo fibular superior (esquerda) e retináculo fibular refletido (direita). ( **B** ) Retinaculoplastia: o retináculo fibular superior é exposto (1) e depois dividido em dois retalhos (2). O retalho proximal é colocado entre os dois tendões fibulares e o retalho distal é colocado superficialmente aos tendões fibulares em posição nativa (3).

**Fig. 4:** Diagrama da técnica cirúrgica.

O sulco fibular é aprofundado preservando a superfície de deslizamento da fibrocartilagem posterior. O retináculo fibular superior é, então, dividido em dois retalhos. O retalho proximal é colocado entre os dois tendões fibulares para evitar o deslocamento intrabainha e reinserido com uma âncora. O retalho distal é colocado superficialmente aos tendões fibulares e reinserido em sua posição nativa para evitar o deslocamento dos tendões fibulares sobre o maléolo lateral ( Figs. 3 e 4 ).

## Desfechos

Ao final do procedimento cirúrgico, deve-se verificar congruência, mobilidade e estabilidade. O paciente é imobilizado com gesso por 3 semanas e depois, com bota imobilizadora por 3 semanas com descarga progressiva de peso.

As 3 pacientes foram avaliadas 2 semanas e 1, 3, 6 e 12 meses após a cirurgia. Na última avaliação, com 1 ano de pós-operatório, todas as pacientes estavam satisfeitas e não apresentavam mais sintomas de subluxação fibular intrabainha. A USD pós-operatória mostrou posicionamento retromaleolar normal dos tendões fibulares tanto em repouso quanto em dorsiflexão ( Fig. 5 ). No entanto, uma paciente (Caso 2) permanece com dor retromaleolar branda e outra (Caso 1) queixa-se de dor no tornozelo (provavelmente uma sequela da fratura).

**Fig. 5:** Ultrassonografia dinâmica pós-operatória: posicionamento retromaleolar normal dos tendões fibulares tanto em repouso (esquerda) quanto em dorsiflexão (direita). Abreviaturas: PL, tendão fibular longo; PC, tendão fibular curto

## Discussão

A instabilidade intrabainha fibular não é comumente relatada e sua incidência exata é desconhecida. 5 Alterações pós-traumáticas podem afetar o retináculo fibular superior ou o sulco fibular. 7 Os pacientes geralmente apresentam histórico de múltiplas lesões por inversão do tornozelo. 7 No Caso 1, a paciente apresentava instabilidade e necessitava de tratamento cirúrgico, o que a predispôs a repetidas entorses de tornozelo devido ao mecanismo de inversão. Isto pode ter contribuído para a consequente instabilidade dos tendões fibulares. No Caso 2, a paciente tinha histórico de entorses de tornozelo recorrentes que também podem ter contribuído para esta lesão. Em todos os casos, as pacientes apresentaram luxação dos tendões fibulares à USD, sem nenhuma ruptura identificada nem na avaliação ultrassonográfica nem à RM; logo, as instabilidades foram classificadas como tipo A.

A RM é bastante útil para avaliação de variações anatômicas ou anomalias intratendíneas, como rupturas de tendões. 3 6 A USD é o melhor estudo de imagem porque permite o exame dos tendões durante sua amplitude de movimento fisiológica. 5 Isso fornece uma avaliação mais precisa de o que ocorre em um paciente com sintomas de subluxação. 6 8

Alguns estudos argumentam que a instabilidade intrabainha dos tendões fibulares é causada por vários fatores que estreitam o túnel fibro-ósseo. Assim, os procedimentos cirúrgicos devem ter como objetivo restaurar o volume desta área anatômica, bem como reconstruir o retináculo fibular superior para prevenir tanto a subluxação intrabainha quanto a luxação anterior dos tendões fibulares. 4 6

Esta técnica é composta pelo aprofundamento posterior do sulco fibular para aumentar a profundidade e área de superfície e por retinuloplastia superior para evitar que os tendões fibulares se desloquem entre si ou em sentido anterior.

## Conclusão

A ultrassonografia dinâmica é essencial e pode revelar uma subluxação intrabainha dos tendões fibulares que de outra forma não seria diagnosticada. O aprofundamento do sulco com retinuloplastia do retináculo fibular superior parece ser um procedimento bem-sucedido nesses pacientes sintomáticos.
